# 2016 Plasma microRNA markers of upper limb recovery following human stroke

**DOI:** 10.1017/cts.2018.176

**Published:** 2018-11-21

**Authors:** Matthew A Edwardson, Xiaogang Zhong, Amrita Cheema, Alexander Dromerick

**Affiliations:** Georgetown – Howard Universities

## Abstract

OBJECTIVES/SPECIFIC AIMS: MicroRNAs are small, non-coding RNAs that control gene expression by inhibiting protein translation. Preclinical studies in rodent stroke models suggest that changes in microRNA expression contribute to neural repair mechanisms. To our knowledge, no one has previously assessed microRNA changes during the recovery phase of human stroke. Our goal was to determine whether patients with significant upper limb recovery following stroke have alteration of neural repair-related microRNA expression when compared to those with poor recovery. METHODS/STUDY POPULATION: Plasma was collected at 19 days post-stroke from 27 participants with mild-moderate upper extremity impairment enrolled in the Critical Periods After Stroke Study. MicroRNA expression was assessed using TaqMan microRNA assays (Thermo Fisher Scientific). Good recovery was defined as ≥6 point change in the Action Research Arm Test (ARAT) score from baseline to 6 months. Bioinformatics analysis compared the plasma microRNA expression profiles of participants with good Versus poor recovery. Candidate biomarkers were identified after correcting for multiple comparisons using a false discovery rate <0.05. RESULTS/ANTICIPATED RESULTS: Eleven microRNAs had significantly altered expression in the good (n=22) Versus poor (n=5) recovery groups, with 2 showing increased expression—miR-371-3p and miR-520g, and 9 showing decreased expression—miR-449b, miR-519b, miR-581, miR-616, miR-892b, miR-941, miR-1179, miR-1292, and miR1296. Three of these could be implicated in neural repair mechanisms. Elevated miR-371-3p levels increase the likelihood that pluripotent stem cells will differentiate into neural progenitors. MiR-892b decreases levels of amyloid precursor protein, which has been implicated as a regulator of synapse formation. Finally miR-941, the only known human-specific microRNA, downregulates the CSPα protein which is involved in neurotransmitter release. DISCUSSION/SIGNIFICANCE OF IMPACT: This preliminary study suggests that circulating microRNAs in the plasma may help serve as biomarkers of neural repair and aid in understanding human neural repair mechanisms. If validated in larger studies with appropriate controls, these markers could aid in timing rehabilitation therapy or designing recovery-based therapeutics.

